# Somatropin therapy in italian adults with growth hormone deficiency

**DOI:** 10.1186/s12902-022-00960-5

**Published:** 2022-03-03

**Authors:** Flavia Pricci, Daniela Rotondi, Marika Villa, Arianna Valerio, Elvira Agazio, Paolo Roazzi

**Affiliations:** 1 Department of Cardiovascular, Endocrine-Metabolic Diseases, and Aging, Istituto Superiore di Sanità, Rome, Italy; 2 National Centre for Health Technology Assessment, Istituto Superiore di Sanità, Rome, Italy

**Keywords:** Growth Hormone, Adulthood, Therapy, Deficit, National Register

## Abstract

**Background:**

In adult population, Growth Hormone Deficiency (GHD) is a complex clinical condition with heterogeneity of causes and duration. Growth Hormone (GH) replacement therapy has beneficial effects entailing a chronic and expensive use. Therefore, entity, appropriateness and standardization of GHD treatment need to be accurately analysed.

In Italy, the epidemiological surveillance on somatropin therapy is entrusted to the National Register of Growth Hormone Therapy (Registro Nazionale degli Assuntori dell’Ormone della Crescita-RNAOC) by the Italian Regulation, in accordance of which the RNAOC-database is collecting the notifications of somatropin prescriptions.

**Methods:**

Aim of this study is to analyse data on somatropin-treated adult population communicated to the RNAOC by the specialist centres of 15 Italian regions and 2 autonomous provinces.

**Results:**

From 2011 to 2019, the somatropin-treated adults were 970 with 4061 examinations (1.21 ± 0.33 visits/year).

The diagnoses were: hypopituitarism (n = 579); hypophysectomy (n = 383); and congenital GHD (n = 3). Five subjects were addressed with diagnoses not included in the regulation.

The starting posology of somatropin was 0.320 (± 0.212) mg/day, 0.292 (± 0.167) mg/day in male and 0.360 (± 0.258) in female patients, with 7 administrations/week in 70.31% of the prescriptions. The differences in posology by gender persisted at 10th year of the follow-up. Starting dosage was higher in patients diagnosed with adult GHD before the age of 30 (0.420 ± 0.225 mg/day), with a progressive decrease of the dosage during the follow-up.

**Conclusions:**

This is the first report on adult GH treatment, describing numbers, diagnoses, and pharmaceutical prescriptions associated to somatropin therapy in a large cohort of Italian GHD-adults.

## Background

In adult population, Growth Hormone Deficiency (GHD) is a syndrome affecting metabolic pathways, adipose and bone tissues, cardiovascular system, etc. Patients could experience increased central adiposity, hyperlipidaemia, and augmented predisposition to atherogenesis, with important impact on the quality of life, including an increased risk of mortality, predominantly due to cardiovascular diseases [1, 4].

It has been demonstrated that GH replacement therapy has beneficial effects on adult GHD. However, it should be considered that this treatment implies a long-term use, with considerable costs and safety concerns. In addition, GHD diagnostic problems have still to be clearly identified.

In this context, reliable epidemiological data on adult GHD are fundamental to measure size, turnover and duration of treatment but they are difficult to obtain due to the heterogeneity of causes and the complexity in diagnosis. In adulthood, GHD could result from the persistency of a child-onset GHD (CO-GHD) or, in the majority of cases, an adult-onset (AO-GHD) condition, with the corresponding difference in pathogenesis, idiopathic or acquired. Another important variable is represented by the diagnostic tests in their application, standardization, and cut-off limits. One more issue is that most of the studies on somatropin treatment are sponsored trials, targeting specific diagnoses or patients.

Given the limited amount of information on growth hormone therapy when applied in the general population, the Italian Ministry of Health at the end of the ‘80s implemented the National Register of Growth Hormone Therapy (Registro Nazionale degli Assuntori dell’Ormone della Crescita-RNAOC). In 2000, the Italian Medicines Agency (AIFA) defined the diagnoses for which somatropin could be reimbursed by the National Health Service (Note#39), with specific criteria for each age category [5–9], and commissioned to the Italian National Institute of Health (Istituto Superiore di Sanità) the collection and analysis of data [10].

Aim of this study is to describe characteristics and therapy in recombinant GH (rGH)-treated adults in the field practice in Italy. The study was conducted on the adult subjects treated with somatropin in 156 centres of 15 Italian regions and 2 autonomous provinces from January 1st 2011 to December 31st 2019.

## Methods

The Italian National Health Service provides adults diagnosed with hypopituitarism, hypophysectomy and congenital GH deficiency, with free-of-charge somatropin. They are diagnosed and treated by the specialists of the clinical centres identified by Italian regions and autonomous provinces [7, 8].

For each treated patient a standard form is to be filled in at each visit and forwarded to the RNAOC at the Italian National Institute of Health.

Since 2011, RNAOC is based on online reporting of rGH prescription forms including a minimum set of mandatory data, i.e. patient personal details, diagnosis and therapy. More details on the web-based RNAOC have already been reported [10].

### Patients and data collection

From January 1st 2011 to December 31st 2019, all information relating to patients aging > 18 with a diagnosis in the group of “adult age” at first visit was selected from the RNAOC database. The following data were considered for each patient: patient’s personal data (date of birth, gender, residence), diagnosis, visits with date of visit, and prescriptions (product, daily posology and weekly frequency of administration).

Patients were followed up until the first of the following events: discontinuation of therapy for any reason, admission to hospital, death. Patients who moved from one clinical centre to another one were considered as treatment continues.

Data underwent quality checks at the data-entry and saving phases, and on the database.

Patients with diagnoses related to the paediatric or the transition age, or not coherent with the chronological age at first visit, were excluded. When subsequent diagnoses were reported, only the first one was considered.

Posology of somatropin therapy was adopted in mg/day. When the weekly frequency of administration was different from 7 days/week, the posology was normalized at mg/day recomputing the total weekly quantity to 7 days.

To calculate the posology according to the year of follow-up for each patient, the follow-up visit closer to the year category was considered.

### Ethic statement

The data collection procedures from clinical units to the RNAOC are in agreement with the Italian legislation for the personal data protection (Legislative Decree 196/2003) and have been updated according to the current European regulation (General Data Protection Regulation, GDPR 2016/679).

Informed consent is not required because processing personal data is essential in order to carry out surveillance activities and drug reimbursement. Anyway, to ensure compliance with the GDPR, an information notice for patients is available in the RNAOC-web platform, with the description of personal data treatment in terms of objective, methods, and ownership.

### Statistical analysis

Statistical analysis was carried out using the STATA software system (StataCorp LLC, TX, USA).

Descriptive statistics, Student’s t-test and ANOVA tests were used to analyse the data. A level of *P* < 0.05 was considered significant.

## Results

At the end of 2019, 15 Italian regions and 2 autonomous provinces had joined RNAOC with 156 clinical centres, and 208 medical outpatients’ facilities indicated as official “prescribing centres”. The accredited clinicians were 350.

During the study period (January 1st 2011 - December 31st 2019), 7332 somatropin treated subjects were reported to RNAOC with some heterogeneity among the regions of the clinical specialist centres.

The subjects older than 18 years with a diagnosis of GHD in the group of “adult age” were 970, 566 (58.35%) males (M) and 404 (41.65%) females (F), representing the 13.23% of all the cases reported to the RNAOC. A total of 4061 visits were carried out and submitted. Discontinuation of treatment was reported in 74 subjects, nine of which were due to death. The frequency of follow-up visits was 1.21 ± 0.33 per year.

In the period 2011–2019, 90.75 ± 75.82 (mean ± SD) adult patients were registered each year and the age at first visit was 48.68 ± 15.60 years (range 18.17–86.89).

Diagnoses in somatropin-treated adults, total and by gender, are reported in Table 1.

**Table 1:** Diagnoses of Growth Hormone Deficiency in adulthood according to AIFA Note#39, as reported to the RNAOC-web platform by the clinical specialist centres from January 1st 2011 to December 31st 2019

Adulthood diagnoses	Subjects		Males		Females		
	**n.**	**%**	**n.**	**%**	**n.**	**%**	
Hypopituitarism: idiopathic, autoimmune hypophysitis, post-cranio-encephalic trauma, surgery or irradiation for sellar or parasellar tumors, empty sella, Sheehan syndrome	579	59.69	338	59.72	241	59.65	
Hypopituitarism: post total or partial hypophysectomy	383	39.48	223	39.40	160	39.60	
Congenital GH deficiency with demonstrated genetic defect	3	0.31	1	0.18	2	0.50	
Not included in Note#39	5	0.52	4	0.70	1	0.25	
**Total**	**970**		**566**		**404**		

These results show that replacement therapy notifications in adult GHD were slightly prevalent in males with no statistically significance between genders. The group of hypopituitarisms not due to surgical ablation was the most frequent cause of adult GHD.

There were 377 subjects with additional pituitary deficiencies: FSH/LH and TSH deficiencies were the most frequent, in 302 (80.11%) and 299 (79.31%) subjects respectively. The pituitary hormone deficiencies were often associated (Table 2).

**Table 2:** Number and percentage of somatropin-treated adults with diagnosis of additional pituitary hormone deficiencies registered to the RNAOC-web platform by the specialists of the clinical centres from January 1st 2011 to December 31st 2019

Pituitary hormone deficiency (n.)	Subjects n. (%)
1	89 (23.61)
2	75 (19.89)
3	149 (39.52)
4	64 (16.98)
**Total**	**377 (100.00)**

For the starting dosage, the mean daily posology at first visit (n = 970) was 0.320 (± 0.212) mg/day, lower in M, 0.292 (± 0.167) mg/day, than in F, 0.360 (± 0.258), (*p* < 0.001), with a frequency of 7 administrations/week in 70.31% of subjects (69.61% in M and 71.29% in F. A percentage of 16.60% of patients received 6 administrations/week.

The initial posology was analysed as related to the diagnosis, showing differences among the causes of GHD and the highest initial dosage in congenital GH deficiency (Table 3).

**Table 3:** Somatropin daily posology (mg/day) prescribed in GHD-adults according to the diagnosis, as reported to the RNAOC-web platform by the clinical specialist centres from January 1st 2011 to December 31st 2019

Adulthood diagnoses	Starting daily posology (mg/day)		5-years daily posology (mg/day)	
	**Mean**	**SD (±)**	**Mean**	**SD (±)**
Hypopituitarism: idiopathic, autoimmune hypophysitis, post-cranio-encephalic trauma, surgery or irradiation for sellar or parasellar tumors, empty sella, Sheehan syndrome	0.337	0.227	0.348	0.266
Hypopituitarism post total or partial hypophysectomy	0.296	0.188	0.304	0.192
Congenital GH deficiency with demonstrated genetic defect	0.424	0.128	-	-
Not included in Note#39	0.206	0.082	0.229	0.049
**All diagnoses**	**0.320**	**0.212**	**0.329**	**0.238**

At 5-year follow-up visit, the daily posology by group of diagnoses does not substantially change. A little, not significant, increment was observed possibly as an effect of the difference in gender compliance.

To analyse the daily posology during the long-term follow-up, all the available visits for each subject were considered.

On 5th year follow-up visits (*n* = 267) the mean posology was 0.329 (± 0.238) mg/day, 0.274 (± 0.138) mg/day in M (*n* = 151) and 0.400 (± 0.311) mg/day in F (*n* = 116) (*p* < 0.001).

**Fig. 1:**
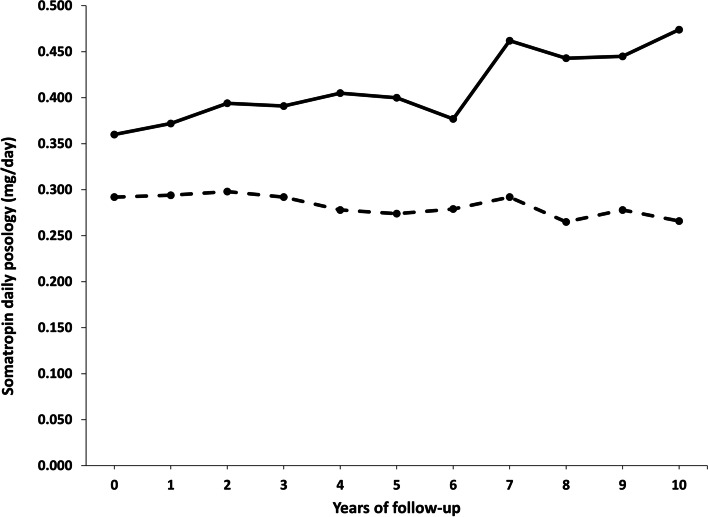
Somatropin daily posology (mg/day) at the follow-up visits of GHD-adults registered in the RNAOC-database by the specialists of the clinical centres from January 1st 2011 to December 31st 2019, gender-disaggregate (- - - Males; ---- Females)

At 10th year follow-up visits (n = 40) the mean daily posology was 0.334 (± 0.252) mg/day, 0.266 (± 0.144) mg/day in M (*n* = 27) and 0.474 (± 0.361) mg/day in F (*n* = 13) (*p* = 0.0125) (Fig. 1).

The diversity in posology by gender was maintained during the follow-up visits with a statistically significant difference all over the follow-up years (Fig. 1; Table 4).

**Table 4:** Counting of somatropin prescriptions at the 10-years follow-up visits gender-disaggregate

Gender	Follow-up visits (year)									
	1	2	3	4	5	6	7	8	9	10
Male	566	318	253	173	133	82	35	23	19	21
Female	405	210	180	136	93	56	28	14	16	12

The daily dosage was also analysed considering the age of subjects at the first administration since guidelines suggest a lower quantity in older patients and a major need in younger patients, especially when they are child-onset GHD. Thus, subjects were divided into 3 age groups: <=30, 31–60 and > 60 years old at first visit.

**Fig. 2:**
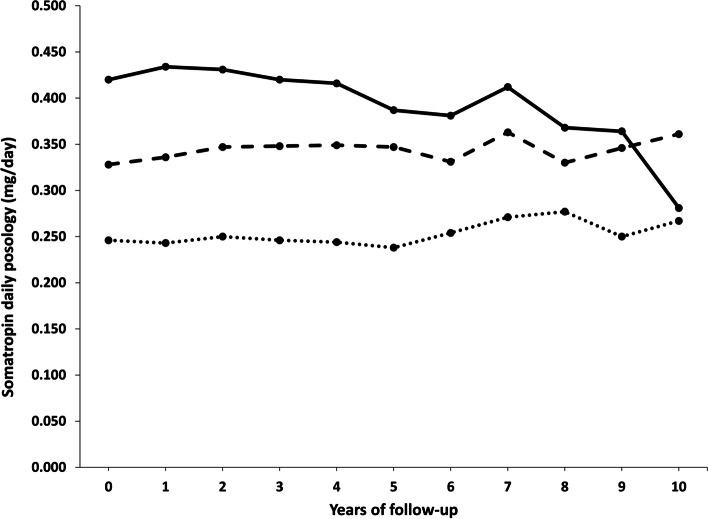
Somatropin daily posology (mg/day) prescribed in GHD-adults registered in the RNAOC database by the clinical specialist centres from January 1st 2011 to December 31st 2019, split according to age groups (---- ≤30; - - - -31-60; …. >60 years old)

According to the recommendations, the starting administered daily posology was lower in the older group (0.246 ± 0.117 mg/day) and higher in the younger one (0.420 ± 0.225 mg/day), with a statistically significant difference among age groups for all the years of the follow-up visits (*p* < 0.001) (Fig. 2; Table 5).

**Table 5:** Counting of somatropin prescriptions at the 10-years follow-up visits split according to age groups

Age (years)	Follow-up visits (year)										
	**0**	**1**	**2**	**3**	**4**	**5**	**6**	**7**	**8**	**9**	**10**
≤ 30	150	150	92	70	50	41	27	18	14	13	10
31–60	574	574	366	298	230	167	100	49	40	34	27
> 60	246	246	134	113	79	59	37	10	5	4	3

The difference between males and females was statistically significant among age groups at 2 years of follow-up. These differences were kept until 5th year in the youngest group, and in the oldest one for 2 years of follow-up. In the intermediate group of age, males and females followed up for 10 years continued to receive statistically different doses.

In detail, in the younger group, the difference between genders was confirmed (F: 0.444 ± 0.237 mg/day vs. M: 0.402 ± 0.216 mg/day), and a progressive decrease of somatropin administration during 10 years of follow-up was observed.

The biosimilar somatropin was prescribed for 84 “naïve” patients at the first visit (9.26%) and 40 patients were “shifted” to the biosimilar product at the follow-up visits (4.12%).

## Discussion

This is the first report from the Italian National Register of Growth Hormone Therapy (RNAOC) on somatropin treatment in adults during the period 2011–2019. Data are communicated by the clinical specialist centres of 15 Italian regions and 2 autonomous provinces, covering approximately 55% of the Italian population (over 32 million people).

Throughout 10 years of data collection, 970 adults treated with somatropin were registered, representing the 13.23% of the rGH prescriptions notified to RNAOC that also collects paediatric and transition age treatments.

The number of subjects reported to the register is not homogeneous among regions probably due to the incomplete adherence of the specialist centres to the mandatory notification of prescriptions, or to local regulations. Similar geographical heterogeneity was observed in the prevalence of somatropin use in a wide cohort of patients from six Italian regions, also evidencing that no rGH consumption pattern could be identified across the Country [11].

Mean baseline age was 48.68 ± 15.60 years, quite similar to that registered in other large surveillance databases containing GHD patients [12–15]. Furthermore, the sex distribution of patients showed that there is no difference between genders, confirming studies from the Dutch National Register study [16] and other European studies based on the epidemiology of hypopituitarism [17, 18]. On the contrary, data obtained from a study combining 3 Danish national registries showed a higher incidence rate in males compared to females in the CO-GHD group and in the AO-GHD group over 45 years of age [19].

These discordances could arise from the heterogeneity of data collections that are based on different criteria, e.g., collection period, specific diagnoses, age of onset, replacement therapy and medicine distribution patterns. In detail, Danish data are the merging of three registries, patients with a cancer diagnosis, the national patient registry, and the cause of death registry from 1980 to 1999, to identify subjects with a diagnosis of GHD using hospitals or city archives [19]. The Dutch register collects patients with severe GHD registered in The Netherlands in the period mid 1998–2008 [16]. Our data are collected on the basis of the Italian regulation established by the authority on medicines that is targeted on pharmacosurveillance rather than on GHD patients and, consequently, the RNAOC database gathers information as required by law with the specific objectives of appropriateness and monitoring of drug consumption [10].

For these reasons, there is a concrete difficulty in comparing data and in estimating the real numbers of adult GHD as well.

According to the AIFA Note#39, adult GHD diagnoses notified to RNAOC are grouped in: hypopituitarism, hypophysectomy, and congenital GH deficiency. The analysis of RNAOC-notified diagnoses shows the prevalence of GHD resulting from hypopituitarism, with no gender differences.

Our data are in line with the evidence that the aetiology of GHD in adults corresponds to the causes of hypopituitarism. In literature, about two-thirds of cases are caused by pituitary tumours or other parasellar masses or resulted from surgery or radiation, and less common causes are traumatic brain injury, granulomatosis, infectious or brain haemorrhages [17, 22, 23]. A small percentage of adult GHD is due to a persistence of GHD in childhood, representing 15–35% of cases in different reports [24, 25]. Moreover, GH is typically the first hormone to become clearly deficient in the majority of the different causes of hypopituitarism [24].

To support these data, about 1/3 of the RNAOC adult somatropin-treated subjects display associated pituitary hormone deficiencies and 56.5% of them has three or more pituitary hormone deficiencies.

GH therapy in adult GHD has been shown to be crucial in supporting morphological, metabolic, physical, and psychological problems. However, there are still some controversies regarding safety, costs, way of administration, and evaluation of benefits. Considering these issues, the replacement regimen has been object of deep evaluation and dosing plans are proposed in guidelines from scientific associations. Individualized treatment is recommended starting with low dose considering the gender, a posology of 0.2 mg/day for men and 0.3 mg/day for women, and the age, in subjects < = 30 years old: 0.4–0.5 mg/day; in adults aged between 31 and 60 years: 0.2–0.3 mg/day; and in subjects > 60 years old: 0.1–0.2 mg/day. Exception is represented by CO-GHD patients who, in general, initially need 50% of the paediatric dosage. Then, a gradual increase of 0.1–0.2 mg/day, if necessary, and an annual assessment should be performed [26–30].

Prescriptions reported in the RNAOC database highlight that the mean initial daily posology of somatropin was slightly higher than suggested by the international recommendations and guidelines, but it becomes aligned when the age groups of patients are considered, demonstrating that the starting doses were adjusted for the age of subjects. Interestingly, the younger rGH treated subjects gradually decreased the daily dosage during the years and reached the posology of the other age groups.

The recommendations to take into account gender are complied with and the differences in dosage are also maintained during the follow-up visits.

Furthermore, the recommendation about the frequency of medical examinations is followed.

One relevant concern for somatropin therapy is about costs that could be reduced by implementing the available somatropin biosimilar. In Italy, the national report on medicine consumption describes a mean of 9.45% of biosimilar somatropin prescriptions with respect to the total rGH preparations in the years 2011–2019, with a progressive increase from 5.5 to 18.2% [31].

In the same years, the prescriptions notified to RNAOC web showed that the adoption of biosimilar somatropin in adults is rather low (13.38%). These data confirm results from a recent extensive study on total rGH consumption in six Italian centres that evidenced a mean of 7.2% of biosimilar rGH users in years 2009–2014, a low percentage compared to other biosimilars (e.g., epoetin alpha and filgrastim), with a variable proportion across different geographical areas. The authors speculate that this variability could be due to the different health-care policies adopted at a loco-regional level, differences in patients’ access to different rGH, different tender procedures for originators and biosimilars purchase by public structures, and the still ongoing scepticism about biosimilars, especially when target patients are paediatric as for rGH [11].

Interestingly, the data on total somatropin biosimilar use are comparable to the results in adult rGH-treated patients registered in RNAOC database.

Definitively, our analysis describes the management of a large Italian cohort of GHD adults treated with somatropin by clinical specialist centres following the regulation by the competent authorities based on updated guidelines indicated by the experts.

The relevance of this report lies in the fact that our data originate from a register appointed for surveillance by national public institutions, and that it can provide complementary information to placebo-controlled, randomized clinical or sponsored trials, by making available epidemiological data, large patient cohorts and pharmaceutical prescriptions. Therefore, the RNAOC database is a useful tool in collecting data from field practice, despite its limits including partial information about treatment efficacy or side effects.

## Conclusions

In conclusion, RNAOC supplies information to the National Health Service, the regional health authorities and the scientific community on numbers, diagnoses, appropriateness, working activity of clinical specialist centres and pharmaceutical consumption related to somatropin therapy in adults.

## Data Availability

RNAOC database is stored at the IT Service of National Institute of Health (Istituto Superiore di Sanità). Data Availability: Restrictions apply to the availability of data generated or analyzed during this study to preserve patient confidentiality or because they were used under license. The corresponding author will on request detail the restrictions and any conditions under which access to some data may be provided.

## Abbreviations

AIFA: Agenzia Italiana del Farmaco-Italian Medicines Agency
AO-GHD: dult-onset Growth Hormone Deficiency
CO-GHD: child-onset Growth Hormone Deficiency
GDPR: General Data Protection Regulation
GH: Growth Hormone
GHD: Growth Hormone Deficiency
rGH: recombinant Growth Hormone
RNAOC: Registro Nazionale degli Assuntori dell’Ormone della Crescita-National Register of Growth Hormone Therapy

## References

[1] Klose UFeldt-Rasmussen,M, Adult Growth Hormone Deficiency Clinical Management. Feingold KR, Anawalt B, Boyce A et al, editors. Endotext [Internet]. South Dartmouth (MA). MDText.com, Inc.; 2000-.

[2] Carroll PV, Christ ER, Bengtsson BA, Carlsson L, Christiansen JS, Clemmons D, Hintz R, Ho K, Laron Z, Sizonenko P, Sönksen PH, Tanaka T, Thorne M (1998). Growth hormone deficiency in adulthood and the effects of growth hormone replacement: a review. Growth Hormone Research Society Scientific Committee. J Clin Endocrinol Metab.

[3] Erfurth EM (2013). Update in mortality in GH-treated patients. J Clin Endocrinol Metab.

[4] Lanes R (2016). Cardiovascular Risk in Growth Hormone Deficiency: Beneficial Effects of Growth Hormone Replacement Therapy. Endocrinol Metab Clin North Am.

[5] Italia. Determinazione dell’Agenzia Italiana del Farmaco 29 ottobre 2004. Note AIFA 2004. Revisione delle Note CUF. Gazzetta Ufficiale n. 259 del 04 novembre 2004, Suppl. Ordinario n. 162.

[6] Italia. Note AIFA 2006–2007 per l’uso appropriato dei farmaci. Gazzetta Ufficiale - Supplemento Ordinario n. 7 del 10 gennaio 2007.

[7] Italia. Determinazione dell’Agenzia del Farmaco 22 settembre 2009. Modifica alla Nota AIFA 39. Gazzetta Ufficiale - Serie Generale n. 238 del 13 ottobre 2009.

[8] Italia. Determinazione dell’Agenzia Italiana del Farmaco 19 giugno 2014. Modifica alla Nota AIFA 39. Gazzetta Ufficiale - Serie Generale n. 154 del 5 luglio 2014.

[9] Italia. Decreto del Presidente del Consiglio dei Ministri del 3 marzo 2017. “Identificazione dei sistemi di sorveglianza e dei registri di mortalità, di tumori e di altre patologie”. Gazzetta Ufficiale - Serie Generale n. 109 del 12 maggio 2017.

[10] Pricci F, Villa M, Maccari F, Agazio E, Rotondi D, Panei P, Roazzi P (2019). The Italian Registry of GH Treatment: electronic Clinical Report Form (e-CRF) and web-based platform for the national database of GH prescriptions. J Endocrinol Invest.

[11] Marcianò I, Ingrasciotta Y, Giorgianni F, Ientile V, Chinellato A, Tari DU, Gini R, Cannavò S, Pastorello M, Scondotto S, Cananzi P, Traversa G, Trotta F, Belleudi V, Addis A, Trifirò G. Pattern of Use of Biosimilar and Originator Somatropin in Italy: A Population-Based Multiple Databases Study During the Years 2009–2014. Front Endocrinol (Lausanne). 2018;9:95. doi: 10.3389/fendo.2018.00095. eCollection 2018. 10.3389/fendo.2018.00095 PMC5859012 29593655

[12] Webb SM, Strasburger CJ, Mo D, Hartman ML, Melmed S, Jung H, Blum WF, Attanasio AF (2009). Changing patterns of the adult growth hormone deficiency diagnosis documented in a decade-long global surveillance database. J Clin Endocrinol Metab.

[13] Abs R, Bengtsson BA, Hernberg-Stahl E, Monson JP, Tauber JP, Wilton P, Wuster C (1999). GH replacement in 1034 growth hormone deficient hypopituitary adults: demographic and clinical characteristics, dosing and safety. Clin Endocrinol.

[14] Gutierrez LP, Koltowska-Haggstrom M, Jonsson PJ, Mattsson AF, Svensson D, Westberg B, Luger A (2008). Registries as a tool in evidencebased medicine: example of KIMS (Pfizer International Metabolic Database). Pharmacoepidemiol Drug Saf.

[15] Attanasio AF, Bates PC, Ho KK, Webb SM, Ross RJ, Strasburger CJ, Bouillon R, Crowe B, Selander K, Valle D, Lamberts SW (2002). Humna growth hormone replacement in adult hypopituitary patients: long-term effects on body composition and lipid status – 3-year results from the HypoCCS Database. J Clin Endocrinol Metab.

[16] Stochholm K, Gravholt CH, Laursen T, Jørgensen JO, Laurberg P, Andersen M, Kristensen L, Feldt-Rasmussen U, Christiansen JS, Frydenberg M, Green A (2006). Incidence of GH deficiency – a nationwide study. Eur J Endocrinol.

[17] van Nieuwpoort IC, van Bunderen CC, Arwert LI, Franken AA, Koppeschaar HP, van der Lelij AJ, Twisk JW, Boers M, Drent ML (2011). Dutch National Registry of GH Treatment in Adults: patient characteristics and diagnostic test procedures. Eur J Endocrinol.

[18] Schneider HJ, Aimaretti G, Kreitschmann-Andermahr I, Stalla GK, Ghigo E. Hypopituitarism. Lancet. 2007;369(9571):1461–1470. 10.1016/S0140-6736(07)60673-4 17467517

[19] Kargi AY, Merriam GR (2013). Diagnosis and treatment of growth hormone deficiency in adults. Nat Rev Endocrinol.

[20] Cook DM, Yuen KC, Biller BM, Kemp SF, Vance ML. American Association of Clinical American Association of Clinical Endocrinologists medical guidelines for clinical practice for growth hormone use in growth hormone-deficient adults and transition patients – 2009 update.Endocr Pract. 2009;15Suppl 2:1–29. doi: 10.4158/EP.15.S2.1. 10.4158/EP.15.S2.1 20228036

[21] Martel-Duguech LM, Jorgensen JOL, Korbonits M, et al. ESE audit on management of Adult Growth Hormone Deficiency in clinical practice. Eur J Endocrinol. 2020: EJE-20-1180.R1. doi: 10.1530/EJE-20-1180. Online ahead of print. 10.1530/EJE-20-1180 33320830

[22] Regal M, Páramo C, Sierra SM, Garcia-Mayor RV (2001). Prevalence and incidence of hypopituitarism in an adult Caucasian population in northwestern Spain. Clin Endocrinol (Oxf).

[23] Sassolas G (1999). GH deficiency in adults: an epidemiological approach. Eur J Endocrinol.

[24] Tanriverdi F, Kelestimur F. Classical and non-classical causes of GH deficiency in adults Best Pract Res Clin Endocrinol Metab. 2017;31(1):3–11. doi: 10.1016/j.beem.2017.02.001. Epub 2017 Feb 23.PMID: 28477730. 10.1016/j.beem.2017.02.001 28477730

[25] Brabant G, Poll EM, Jonsson P (2009). Etiology, baseline characteristics, and biochemical diagnosis of GH deficiency in the adult: are there regional variations?. Eur J Endocrinol.

[26] Molitch ME, Clemmons DR, Malozowski S, Merriam GR, Vance ML (2011). Endocrine Society. Evaluation and treatment of adult growth hormone deficiency: an Endocrine Society clinical practice guideline. J Clin Endocrinol Metab.

[27] Yuen KCJ, Biller BMK, Radovick S, Carmichael JD, Jasim S, Pantalone KM, Hoffman AR (2019). American Association of Clinical Endocrinologists and American College of Endocrinology guidelines for management of growth hormone deficiency in adults and patients transitioning from paediatric to adult care. Endocr Pract.

[28] Ken KY, Ho. 2007 GH Deficiency Consensus Workshop Participants. Consensus guidelines for the diagnosis and treatment of adults with GH deficiency II: a statement of the GH Research Society in association with the European Society for Pediatric Endocrinology, Lawson Wilkins Society, European Society of Endocrinology, Japan Endocrine Society, and Endocrine Society of Australia. Eur J Endocrinol 2007;157(6):695–700. 10.1530/EJE-07-0631 18057375

[29] Gasco V, Prodam F, Grottoli S, Marzullo P, Longobardi S, Ghigo E, Aimaretti G. GH therapy in adult GH deficiency: a review of treatment schedules and the evidence for low starting doses Eur J Endocrinol 2013;168(3):R55-66. doi: 10.1530/EJE-12-0563. Print 2013 Mar. 10.1530/EJE-12-0563 23152440

[30] Boguszewski CL. Update on GH therapy in adults F1000Res. 2017;6:2017. 10.12688/f1000research.12057.1 PMC5691372 29225782

[31] Italian Medicines Agency. National Report on Medicines Use in Italy. Available from: https://www.aifa.gov.it/en/rapporti-osmed.

